# From soil to crop: a tiered screening strategy for lipopeptide-producing *Bacillus* with antifungal, antibacterial and plant growth-promoting activities

**DOI:** 10.3389/fmicb.2026.1798265

**Published:** 2026-04-28

**Authors:** Tian Gan, Tingying Li, Huimin Yu, Aijia Yu, Mengyao Zhao, Chendi Zhao, Lishuang Xu, Jiaao Cheng, Yuchao Ma

**Affiliations:** 1 College of Biological Sciences and Technology, Beijing Forestry University, Beijing, China; 2 Department of Chemical Engineering, Tsinghua University, Beijing, China; 3 College of Forestry, Beijing Forestry University, Beijing, China

**Keywords:** antibacterial and antifungal activities, genome mining, lipopeptide-producing *Bacillus*, plant growth promotion, tiered screening

## Abstract

**Introduction:**

To address agrochemical pollution and improve microbial screening efficiency, this study aimed to develop an integrated pipeline for isolating high-yielding lipopeptide-producing *Bacillus* strains and validating their agricultural applications.

**Methods:**

A tiered screening strategy combining hemolytic activity assay, plate confrontation, and oil-spreading assay was established to isolate strains from rhizosphere soils. Lipopeptide production was identified by MALDI-TOF MS and quantified via HPLC. Genomic analysis was performed to elucidate the genetic basis of lipopeptide synthesis. Crude lipopeptide extracts were evaluated for antimicrobial activity via plate assays, and for plant growth-promoting effects under pot and field conditions.

**Results:**

The screening successfully identified potent *Bacillus* producers with multiple plant growth-promoting traits. Strain A1C-8 produced 1140.7 mg/L surfactin, Bv1 produced 450.1 mg/L iturin, and C5C-6 produced 707.8 mg/L fengycin. Crude extracts exhibited strong antimicrobial activity, with inhibition rates of 54.6% against *Staphylococcus aureus* and 273.3% against *Rhizoctonia solani*. Genomic analysis confirmed a robust genetic basis for lipopeptide synthesis. Notably, field trials demonstrated that lipopeptide extracts alone significantly enhanced crop growth: maize ear fresh weight increased by 32.46 % under 100 mg/L treatment, peanut pod number per plant rose by 42.75 % under 200 mg/L treatment, and sunflower fresh seed weight per capitulum improved by 35 % with 50 mg/L application.

**Discussion:**

This study provides an integrated pipeline from strain screening to field validation, highlighting Bacillus lipopeptides as sustainable alternatives to agrochemicals for combined biocontrol and plant growth promotion.

## 1 Introduction

By 2050, the global population is projected to reach approximately 10 billion, necessitating a substantial increase in food production, a challenge further exacerbated by climate change, which compels an urgent improvement in crop yields (Sandhu et al., 2025). Concurrently, malnutrition and obesity highlight deficiencies in food quality and diversity (Kiosia et al., 2024). Since agricultural expansion exacerbates greenhouse gas emissions and biodiversity loss, sustainable intensification must focus on improving productivity per unit area (Pradhan et al., 2015). Microbial-based technologies, which enhance nutrient acquisition and protect crops from soil-borne pathogens, offer a promising low-input strategy for sustainable agriculture (Lastochkina et al., 2022). Their effectiveness, however, relies on the presence of beneficial rhizosphere consortia capable of sustainably producing bioactive metabolites under field conditions (Rawte and Saxena, 2025). Consequently, systematic mining of rhizosphere microbiomes for potent biosynthetic strains is crucial for developing effective bioinoculants.

The prolonged use of traditional chemical pesticides leads not only to environmental and agricultural problems, such as soil degradation and pest resistance, but also to direct human health risks, including acute conditions like skin irritation and poisoning (Tomar et al., 2024). In response, microbial control agents, such as specific strains of *Bacillus* and *Pseudomonas*, offer a sustainable alternative by synthesizing diverse bioactive compounds—including antibiotics, siderophores, and phytohormones—that suppress pathogens while simultaneously enhancing plant growth and nutrient uptake (Daraban et al., 2023). A global meta-analysis confirms that biological treatments significantly improve crop performance with yield increases up to 21% (Lamichhane et al., 2022). Such targeted, multifunctional biopesticides align with organic agriculture goals emphasized in the EU’s 2030 Agenda (Pânzaru et al., 2023).

The genus *Bacillus* is renowned for producing diverse secondary metabolites, particularly lipopeptides such as surfactin, iturin, and fengycin (Valenzuela Ruiz et al., 2024). Surfactin shows strong surface activity and antibacterial effects (Abdelraof et al., 2024), while iturin and fengycin show antifungal activity by disrupting the integrity of microbial cell membranes, leading to cell death (Dini et al., 2024). In crops, such as chili pepper and lettuce, they have been shown to significantly increase biomass and germination (Umar et al., 2021). Moreover, beyond this direct mode of action, these lipopeptides can also enhance plant defense by activating jasmonic acid and salicylic acid signaling pathways (He et al., 2024) and contribute to durable disease suppression by altering the rhizosphere microbiome to enrich beneficial microorganisms (Zhao et al., 2025). Beyond agriculture, lipopeptides have applications in medicine, environmental remediation, and food preservation (Sani et al., 2024). In medical research, iturin A has demonstrated significant antitumor activity against hepatocellular carcinoma by inducing multiple cell death pathways and improving the tumor immune microenvironment. Biosynthetically, lipopeptides are assembled by non-ribosomal peptide synthetases (NRPSs), through complex, modular pathways (Ishikawa et al., 2024). However, wild-type strains typically produce only several hundred mg/L, limiting industrial scalability (Coutte et al., 2017). For example, *Bacillus velezensis* UTB96 produced only approximately 140 mg/L surfactin, 620 mg/L iturin A, and 45 mg/L fengycin in a 45-liter bioreactor, but already outperformed the model strain *B. velezensis* FZB42 (Vahidinasab et al., 2022). Therefore, screening for high-yielding lipopeptide-producing strains and optimizing their production are critical for commercial application.

Current screening methods for lipopeptide producers largely rely on physicochemical assays detecting general biosurfactant activity, such as the drop collapse test, Du-Nouy ring technique, and oil displacement assay (Mnif and Ghribi, 2015). These methods often yield false positives and do not quantitatively link antimicrobial activity to lipopeptide yield or composition (Biniarz et al., 2017). Moreover, most screening focuses narrowly on antagonism, overlooking other plant-beneficial traits, which limits the discovery of multifunctional biocontrol agents (Williams et al., 2025). A further disconnect between preliminary screening and genetic validation hinders the efficient identification of novel strains with both high lipopeptide production and robust application potential.

The rhizosphere represents a rich reservoir of beneficial bacteria capable of synthesizing diverse biocontrol metabolites (Thepbandit and Athinuwat, 2024). In this study, we collected rhizosphere soil samples to implement a multi-tiered screening strategy for high-yield lipopeptide-producing *Bacillus* strains. Initial selection was based on hemolytic activity, followed by antagonism assays, and oil displacement tests. Lipopeptide yield and composition were correlated with antagonistic activity. Promising isolates were further evaluated for plant growth-promoting rhizobacterial (PGPR) traits and subjected to whole-genome sequencing and NRPS cluster analysis to confirm their biosynthetic potential. This integrated approach bridges functional screening with genetic characterization, enabling the identification of multifunctional strains with high lipopeptide production and robust antagonistic activity. Moreover, the plant growth-promoting effects of the selected strains and their lipopeptides were validated in both pot and field trials across multiple crops, providing a solid foundation for sustainable agricultural applications.

## 2 Materials and methods

### 2.1 Isolation, screening and identification of lipopeptide-producing *Bacillus*

#### 2.1.1 Sample collection and bacterial isolation

Soil samples were collected from 14 different cities throughout China (Supplementary Table S1). Rhizosphere soil was collected at a depth of 20 cm, sealed in sterile plastic bags, and transported to the laboratory for microbiological analysis.

#### 2.1.2 A tiered screening strategy for lipopeptide-producing *Bacillus*

The process began with the isolation of bacteria from a soil suspension, followed by heat treatment at 80 °C for 10 min, serial dilution, and plating on sheep blood agar. After incubation at 37 °C for 2 days, primary selection was based on the formation of large transparent hemolysis circles. Promising strains were then evaluated in a plate confrontation assay against *Rhizoctonia solani*, where a mycelial block and a bacterial spot were inoculated 2.0 cm apart on PDA. Antifungal activity was quantified by measuring the inhibition zone width after 5 days at 25 °C. Finally, a film was formed by spreading 200 μL of 0.5% Oil Red O in n-dodecane on water, to which 2.5 μL of fermentation supernatant was added. The diameter of the resulting clear zone was measured to estimate lipopeptide concentration. The reliability of this method was validated by a standard curve prepared with surfactin standard (MCE, purity 98%).

#### 2.1.3 Molecular identification and phylogenetic analysis

For taxonomic identification of the bacterial isolates, 16S rRNA genes were amplified and sequenced using the primers 27F (5′-AGAGTTTGATCCTGGCTCAG-3′) and 1492R (5′-TACGGCTACCTTGTTACGACTT-3′). Multiple sequence alignments were generated with the 16S rRNA sequences of antagonistic strains, and available sequences of related species were downloaded from the NCBI databank. A phylogenetic tree was constructed using the MEGA 11 software based on the Neighbor-joining method. Numbers at branching points represent bootstrap values from 1,000 replications; only values ≥50% are shown. Accession numbers are provided in parentheses.

### 2.2 Extraction and antimicrobial activity of lipopeptides

#### 2.2.1 Extraction of lipopeptides from *Bacillus* spp.

Lipopeptides were extracted from *Bacillus* spp. fermentation via acid precipitation (Romano et al., 2011). For antagonism assays, strains were grown in triplicate in 30 mL of fermentation medium (soluble starch 1.0 g, glucose 15.0 g, soybean powder 30.0 g, yeast extract 0.2 g, KH_2_PO_4_ 1.5 g, K_2_HPO_4_ 3.0 g, MgSO_4_·7H_2_O 0.5 g, CaCO_3_ 0.1 g, FeSO_4_ 0.1 g, distilled water 1,000 mL) at 34 °C, 180 rpm for 72 h. After fermentation, 20 mL of the fermentation supernatant was used for crude lipopeptide extraction by acid precipitation. The precipitate was then dissolved in 3 mL of methanol, adjusted to pH 7.0 with NaOH, brought to a final volume of 6.5 mL with PBS, and finally filtered through a 0.22 μm membrane. For plant growth-promotion experiments, *B. velezensis* Bv1 was cultivated in a 200 L bioreactor at 37 °C for 42 h under controlled conditions (200 rpm agitation and 0.1 vvm aeration). The resulting precipitate was collected by acid precipitation, neutralized to pH 7.0 with 2 M NaOH, and lyophilized (LGJ-200F, Songyuan Huaxing, China).

#### 2.2.2 Antibacterial and antifungal activity assays

The antibacterial and antifungal activities of lipopeptide extracts were evaluated using a well diffusion assay. For the antibacterial assay against *Staphylococcus aureus*, molten LB agar was mixed with bacterial suspension (OD_600_ = 1.0) at 1:100 (v/v) prior to plate pouring. For the antifungal assay against *R. solani*, a 5 mm mycelial plug was placed at the center of a fresh PDA plate. In both assays, four equidistant wells were created around the center. Three wells were filled with the lipopeptide crude extract, while the fourth received a PBS-methanol control. The sample volume was 100 μL for *S. aureus* and 200 μL for *R. solani*. After incubation at 37 °C overnight for bacteria or 25 °C for 7 days for fungi, the inhibition rate was calculated using Equation (1) for *S. aureus* and *R. solani*.


Inhibition rate(%)=[(D−d)/d]×100 (1)

where *D* is the total diameter of the inhibition zone, and *d* is the diameter of the well.

### 2.3 Chemical characterization of lipopeptides

#### 2.3.1 Lipopeptide profiling by MALDI-TOF MS

Lipopeptides in the culture supernatants were analyzed by MALDI-TOF MS on an AXIMA Performance™ (Shimadzu, Japan) equipped with a 337 nm nitrogen laser for desorption/ionization. An equal volume of 0.1% α-cyano-4-hydroxycinnamic acid was used as the matrix in a matrix-to-analyte ratio between 1:1 and 10:1. Spectra were acquired in reflection mode with an accelerating voltage of 20 kV, accumulating 50 laser shots per spectrum (Yang et al., 2015). The m/z range was set from 500 to 2,000 for molecular mass determination.

#### 2.3.2 Accurate mass measurement of lipopeptides by HPLC

To determine the concentration of three types of lipopeptides, fermentation supernatant was filtered through a 0.2 μm PES membrane filter. Lipopeptide concentrations were determined with a Shimadzu LC-20A HPLC system (Kyoto, Japan) fitted with an Inertsustain C18 column (4.6 mm × 250 mm, 5 μm). For all analyses, the injection volume was 20 μL. Individual standards of surfactin, iturin, and fengycin (MCE, purity ≥98%) were used to establish calibration curves. For surfactin, the mobile phase was methanol/0.1% TFA (90:10, v/v) with a flow rate of 0.8 mL/min and detection at 215 nm. Iturin was analyzed using 0.035% formic acid/acetonitrile (55:45, v/v) at 1.0 mL/min with detection at 220 nm, while fengycin was quantified with 0.1% formic acid/acetonitrile (48:52, v/v) at 0.8 mL/min and detection at 205 nm.

### 2.4 Detection of bacterial traits for plant health

For indole-3-acetic acid (IAA) quantification, single colonies were cultured in LB broth containing 100 mg/L L-tryptophan. The IAA production was determined by the Salkowski reagent, and the absorbance was measured at 530 nm using a spectrophotometer (Glickmann and Dessaux, 1995). The concentration of IAA was determined from a standard curve of purified IAA (BioDee, China, purity ≥98%). To screen for siderophore production, bacterial cultures grown overnight in LB medium were spot-inoculated on CAS plates, and the plates were incubated at 30 °C for 4 days (Schwyn and Neilands, 1987). The isolates exhibiting an orange halo were considered positive for siderophore production. Phosphate solubilization ability was evaluated on Pikovskaya’s agar medium (Pikovskaya, 1948). The inoculated plates were kept for 4 days at 28 °C. The appearance of clear zone by the colonies was considered to indicate phosphate solubilizing capacity. Extracellular protease activity was assayed by a modification method of Mehrotra et al. (1999). Bacterial strains were spot-inoculated onto skim milk agar, and the clear zones around the colonies indicated casein hydrolysis and soluble nitrogen release.

### 2.5 Genomic sequencing and bioinformatics analysis

#### 2.5.1 Whole genome sequencing and annotation

Genomic DNA was extracted and purified using the Wizard® Genomic DNA Purification Kit (Promega), followed by quantification with a TBS-380 fluorometer (Turner BioSystems Inc., Sunnyvale, CA). Whole-genome sequencing was performed by Shanghai Majorbio Bio-pharm Technology Co., Ltd. using a combined PacBio RS II SMRT and Illumina platform. Gene prediction was conducted with Glimmer (CDS), tRNA-scan-SE (tRNA), and Barrnap (rRNA). The predicted coding sequences were functionally annotated against the NR, Swiss-Prot, Pfam, GO, COG, and KEGG databases using BLAST, Diamond, and HMMER.

#### 2.5.2 Pan-genome analysis of *Bacillus velezensis*

Genomic sequences of 12 *B. velezensis* strains, comprising three newly sequenced and nine published antagonistic isolates, were uniformly annotated via the Prokka pipeline on the Majorbio Cloud Platform (Han et al., 2024). Pan-genome analysis was performed using the platform’s PGAP workflow with the gene family model, applying thresholds of ≥50% amino acid identity, ≥50% coverage, an *E*-value of ≤1 × 10^−5^, and an MCL inflation parameter of 1.5 to define orthologous clusters. Core, accessory, and strain-specific gene sets were extracted from the resulting matrix. Core genes were functionally categorized by BLASTp against the COG database, while pan- and core-genome accumulation curves were generated using the PanGP module with 1,000 random permutations.

#### 2.5.3 In-depth genomic characterization

The genomic species delineation of the isolate was confirmed by calculating the Average Nucleotide Identity (ANI) against closely related type strains using the JSpecies Web Server (Richter et al., 2016) with the ANIm algorithm. The surfactin (*srfA*), iturin (*itu*), and fengycin (*fen*) gene clusters were identified using antiSMASH and validated against the MIBiG database. Subsequently, the domain architectures of their core biosynthetic proteins were predicted using the InterPro Scan program.

### 2.6 Evaluation of plant growth promotion by lipopeptides in pot and field

#### 2.6.1 Effects on maize: from pot to field

A pot experiment was conducted at Beijing Forestry University (40.01°N, 116.34°E) to examine the effects of *Bacillus* spp. and its extracts on maize. Using a completely randomized design with four replicates, pots were sown with the maize cultivar Jiyuan-128. One week after sowing, seedlings were root-irrigated with 200 mL per pot of either lipopeptide extract at 50 mg/L, a suspension of lyophilized bacterial powder at 500 mg/L, or water as control, applied to different groups. Plants were harvested 10 and 20 days after treatment for parameter measurement.

A complementary field experiment was performed at the Dongsheng Experimental Primary School in Haidian District (40.01°N, 116.34°E) using the maize variety Jingke Sweet 608. Eight days after sowing, seedlings were soil-drenched with lipopeptide solutions at 50, 100, or 200 mg/L, with water as control. Plant height was measured at the jointing stage, and yield-related traits were recorded per ear at the milk-ripening stage.

#### 2.6.2 Optimization and field evaluation on peanut

A two-phase field experiment was conducted to evaluate lipopeptides in peanut cultivation. The first phase for concentration screening was carried out at Beijing Forestry University. Peanut seedlings at 21 days after sowing were soil-drenched with 200 mL per plant of lipopeptide solutions at concentrations of 10, 50, 200, 500, or 1,000 mg/L, using water as a control. Upon identification of the optimal concentration at 200 mg/L, the second validation phase was implemented at Dongsheng Primary School, applying the same volume of the 200 mg/L solution per plant versus a water control at the seedling stage.

Common field management practices were followed. Pods from both phases were harvested, rinsed, air-dried to constant weight, and assessed for yield. Additionally, in the validation phase, pod quality traits including double-pod rate, plump kernel rate, 100-pod weight, and 100-kernel weight were determined.

#### 2.6.3 Field assessment on sunflower

A field experiment was performed at Beijing Forestry University to assess the growth-promoting effects of lipopeptides on sunflower. Plants were maintained under standard field conditions with irrigation and weeding applied as required. Plant height and stem diameter were measured at 24 days after treatment. Sunflowers were harvested at physiological maturity, which occurred at 92 days after treatment, and capitulum diameter was recorded. The fresh seed weight per capitulum was determined immediately following harvest.

### 2.7 Data analysis

Data are presented as mean ± SD from at least three independent replicates. Statistical analyses were performed using SPSS 27.0 (SPSS Inc., Chicago, IL, United States). Differences among groups were assessed by one-way ANOVA, and a *p*-value of less than 0.05 was considered statistically significant.

## 3 Results

### 3.1 A tiered screening strategy identifies high-yielding lipopeptide-producing *Bacillus* strains

**Figure 1:**
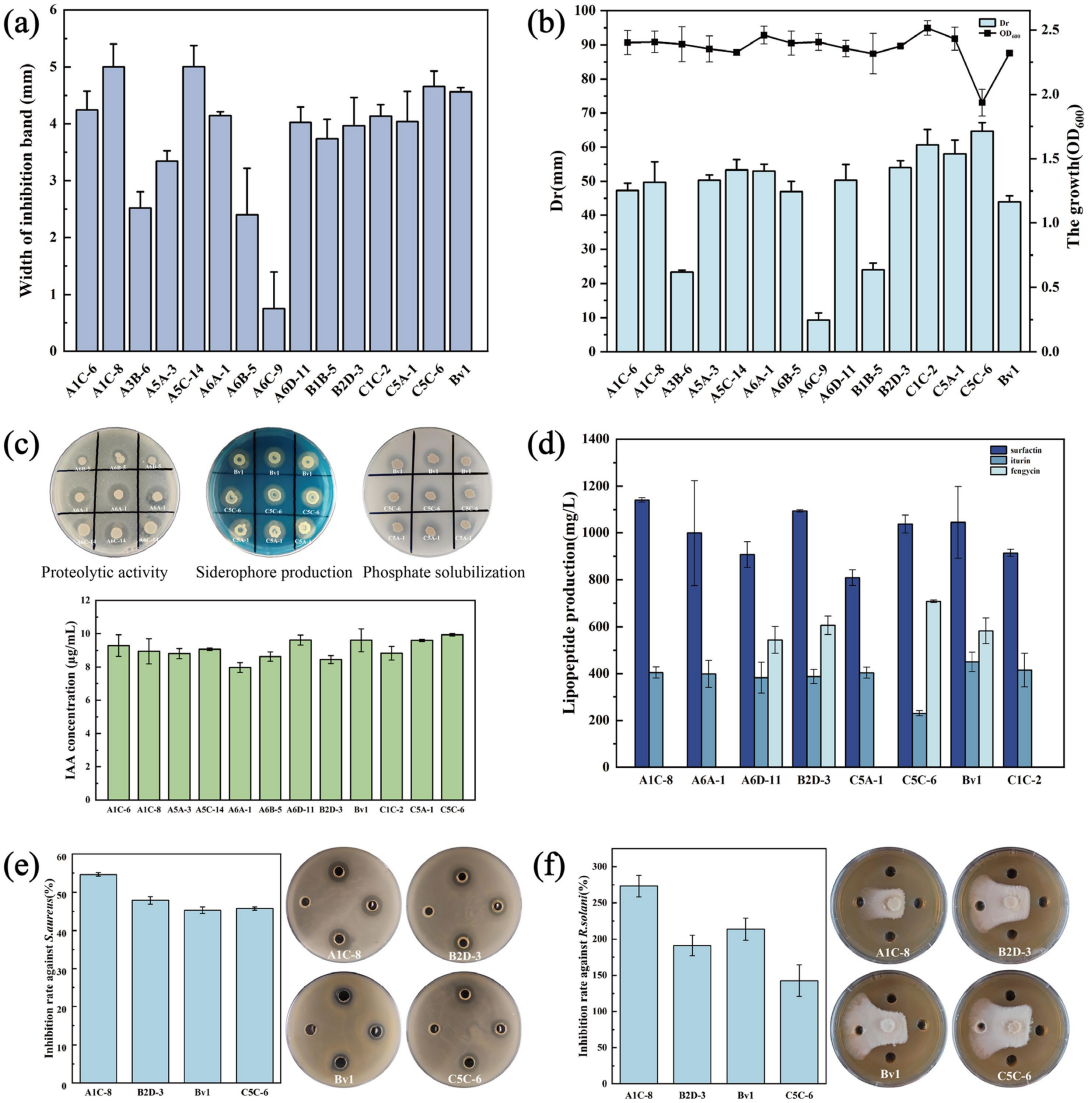
Comprehensive *in vitro* screening and characterization of lipopeptide-producing *Bacillus* isolates. **(a)** Inhibition zone widths against *R. solani* measured in mm. **(b)** Lipopeptide production (Dr, mm) and culture density (OD_600_). **(c)** Plant growth-promoting traits. **(d)** Lipopeptide production by HPLC analysis. **(e)** Antimicrobial activity against *S. aureus*. **(f)** Antimicrobial activity against *R. solani*.

In this study, we devised and implemented a sequential, three-tiered functional screening strategy targeting native *Bacillus* strains from diverse plant rhizospheres (Figure 1; Supplementary Table S1). This strategy was designed to filter isolates based on lipopeptide-linked phenotypes, ensuring the initial candidates possessed high production capacity and core biocontrol function. These selected strains then serve as a qualified pool for subsequent evaluation of plant growth-promoting potential.

The first tier utilized hemolytic activity on sheep blood agar as an initial screening proxy for lipopeptide production. Consequently, the appearance of a hemolytic zone serves as a rapid and indicative phenotype for the potential production of these lipopeptides by *Bacillus* strains. From over 100 hemolytic isolates, the second tier of selection was based on direct antagonism against the devastating soil-borne fungus *R. solani*, thereby identifying strains capable of synthesizing antifungal metabolites. This step identified 29 antagonistic strains, 15 of which formed clear inhibition zones, confirming the presence of bioactive compounds (Figure 1a; Supplementary Figure S1). The third and quantitative tier employed an oil-spreading assay, calibrated with pure surfactin, on these 15 strains. This step was crucial as it moved beyond qualitative antagonism to quantify biosurfactant production, a direct indicator of amphiphilic lipopeptide yield. A strong linear correlation (*R*^2^ > 0.98) between halo diameter and surfactin concentration in the range of 400 to 1,400 mg/L validated the reliability of the assay for rapid semi-quantification (Supplementary Figure S2). This final funnel identified 12 elite isolates forming halos >40 mm, with C5C-6 exhibiting the largest diameter of 64.67 mm (Figure 1b), indicating their potential for high lipopeptide production.

**Figure 2:**
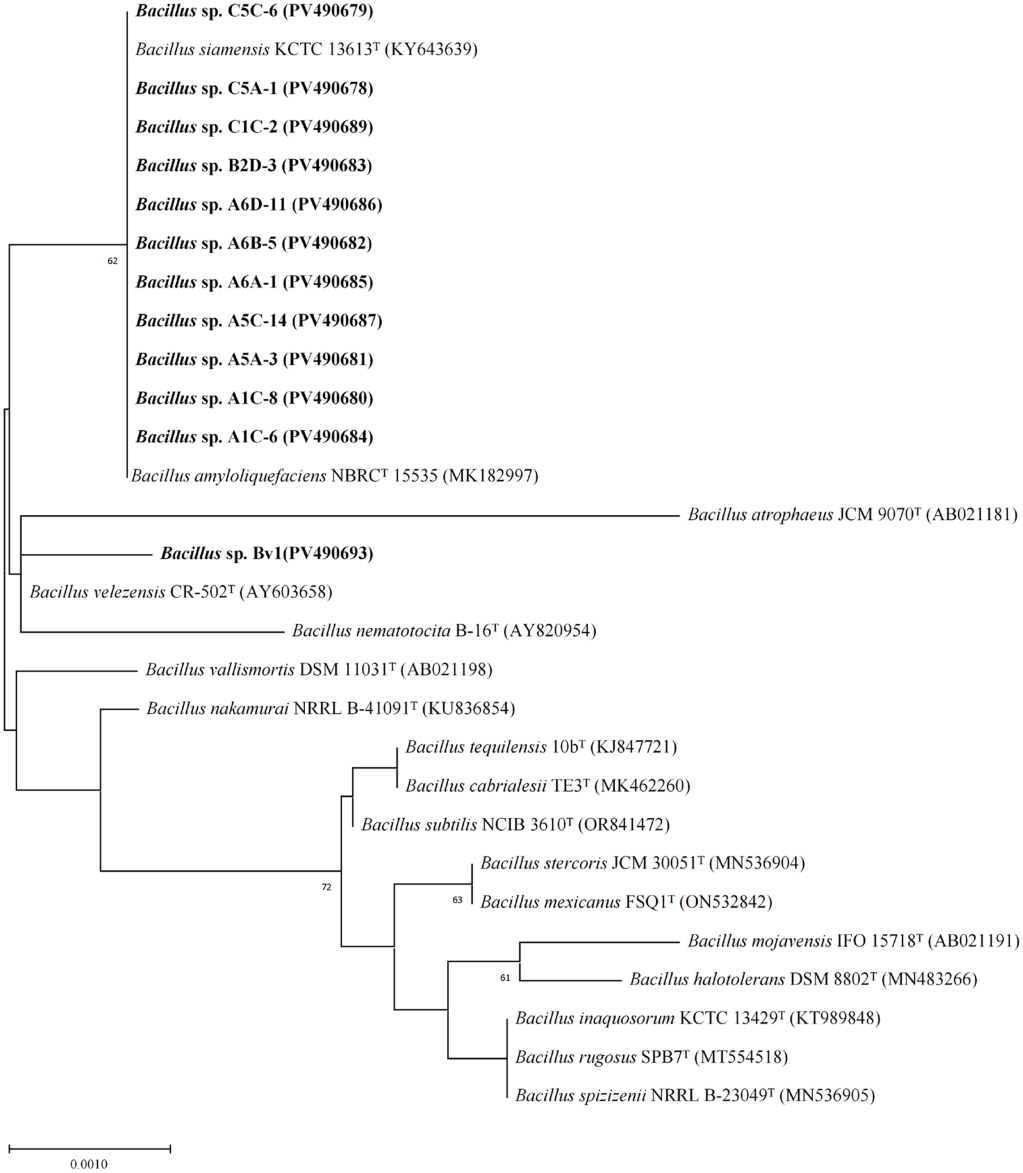
Neighbor-joining tree based on 16S rRNA gene sequences showing the phylogenetic positions of isolates compared to the closely related members of the genus *Bacillus*. Numbers at branching points represent bootstrap values from 1,000 replications; only values ≥50% are shown. Accession numbers are given in parentheses.

The 12 antagonistic strains obtained from the screening were subjected to molecular identification based on 16S rRNA gene sequencing. Phylogenetic analysis using the neighbor-joining method revealed that all these strains belonged to the genus *Bacillus* (Figure 2). Eleven strains grouped together with *Bacillus amyloliquefaciens* NBRC 15535 and *Bacillus siamensis* KCTC 13613. Strain Bv1 did not cluster with the other isolates, but formed a distinct clade that was most closely related to *B. velezensis* CR-502 and *Bacillus nematocida* B-16, and further affiliated with *Bacillus atrophaeus* JCM 9070.

### 3.2 Elite isolates exhibit multiple plant growth-promoting traits essential for rhizosphere competence

*Bacillus* amphiphilic cyclic lipopeptides (CLPs) are prominent chemical mediators of ecological interactions. However, relying solely on such single traits frequently leads to inconsistent field performance. Therefore, we comprehensively profiled the 12 high-yielding lipopeptide *Bacillus* strains for key PGPR traits: siderophore production, indole-3-acetic acid (IAA) synthesis, phosphate solubilization, and protease activity (Figure 1c; Supplementary Table S2). Notably, all strains exhibited a multifaceted PGPR profile, which is a significant advantage for developing stable inoculants.

Overall, the 12 strains exhibited a spectrum of PGPR traits, with strains C5A-1, C5C-6, and Bv1 consistently ranking among the top performers across multiple assays. All tested bacterial strains produced IAA at concentrations ranging from 7.97 to 9.94 mg/L, with strains C5C-6 and Bv1 exhibiting the highest yields of 9.94 and 9.61 mg/L, respectively (Supplementary Table S2). Siderophore production was detected in all 12 strains via yellow halos around the colony on CAS agar, with C5A-1 showing the strongest ability (Figure 1c). For phosphate solubilization, isolate C5A-1 displayed the highest solubilization index, followed by Bv1 and C5C-6, whereas A5A-3 showed the lowest (Figure 1c). Additionally, all strains exhibited significant proteolytic activity and formed clear zones of protein hydrolysis, with the exception of A6B-5 (Figure 1c).

### 3.3 Lipopeptide profiling and quantification reveal high-yield producers

Lipopeptides are a diverse group of microbial natural products. They consist of a hydrophobic fatty acyl chain linked to a hydrophilic, often cyclized, peptide portion of CLPs. To precisely characterize the metabolic output of the shortlisted strains, we employed both qualitative profiling by MALDI-TOF MS and absolute quantification by HPLC. This two-pronged analytical approach confirmed the success of our screening strategy and revealed distinct lipopeptide production phenotypes among the elite isolates.

Eight isolates were selected based on an oil displacement circle diameter (Dr) exceeding 40 mm and beneficial PGPR traits for subsequent lipopeptide analysis. This analysis, performed using MALDI-TOF MS, provided a detailed molecular snapshot, detecting homologs from all three major families: surfactin, iturin and fengycin, across different strains (Supplementary Figure S3; Table 1). Strains A1C-8, A6A-1, C5A-1, and C1C-2 were found to produce surfactin A (m/z 1,000–1,100) and iturin A (m/z 1,040–1,140). In addition to surfactin A and iturin A, strains A6D-11, B2D-3, C5C-6, and Bv1 also produced fengycin A (m/z 1,400–1,600). For surfactin A, homologs with C13-C15 fatty acid chains were detected in all strains except C5A-1, which lacked the C13 homolog. The predominant peaks corresponded to potassium adducts [M + K]^+^ at m/z 1,047 (C13), 1,061 (C14), and 1,075 (C15). For iturin A, strain C5C-6 produced four iturin A homologs with C14-C18 fatty acid chains, whereas the remaining strains produced one or two homologs within the same range. Characteristic peaks included potassium adducts [M + K]^+^ at m/z 1,081 (C14), 1,095 (C15), and 1,123 (C17), a potassium-water adduct [M + K + H2O]^+^ at m/z 1,113 (C15), and both protonated [M + H]^+^ and potassium adduct [M + K]^+^ species at m/z 1,099 and 1,137, respectively (C18). For fengycin A, strain B2D-3 produced homologs with C14-C18 fatty acid chains, whereas the other three strains produced two to four homologs within this range. Major peaks included protonated [M + H]^+^ and potassium adduct [M + K]^+^ species at m/z 1,435 and 1,473 (C14), 1,449 and 1,487 (C15), sodium and potassium adducts [M + Na]^+^ and [M + K]^+^ at m/z 1,485 and 1,501 (C16), and [M + Na]^+^ and [M + K]^+^ at m/z 1,499 and 1,515 (C17), as well as [M + K]^+^ at m/z 1,529 (C18). This diversity is functionally important, as homolog chain length influences critical properties like critical micelle concentration, membrane fluidity interaction, and ultimately, antimicrobial potency.

**Table 1:** Identification of lipopeptides in different bacterial strains by MALDI-TOF MS.

Lipopeptide type	A1C-8	A6A-1	A6D-11	B2D-3	C5A-1	C5C-6	Bv1	C1C-2
C_13_ Surfactin A	+	+	−	+	−	+	+	+
C_14_ Surfactin A	+	+	+	+	+	+	+	+
C_15_ Surfactin A	+	+	+	+	+	+	+	+
C_14_ Iturin A	−	−	−	−	−	+	+	−
C_15_ Iturin A	−	+	−	−	−	+	+	−
C_17_ Iturin A	+	−	−	−	−	+	−	+
C_18_ Iturin A	+	−	+	+	+	+	−	−
C_14_ Fengycin A	−	−	−	+	−	−	+	−
C_15_ Fengycin A	−	−	−	+	−	−	+	−
C_16_ Fengycin A	−	−	+	+	−	+	+	−
C_17_ Fengycin A	−	−	−	+	−	−	+	−
C_18_ Fengycin A	−	−	+	+	−	+	−	−

Quantitative HPLC analysis translated these profiles into tangible production metrics, revealing high yields (Figure 1d). Surfactin was the dominant lipopeptide in most strains, with A1C-8 reaching an exceptionally high titer of 1,140 mg/L, a yield that rivals some engineered strains and is highly promising for industrial consideration. Strain Bv1 was the premier producer of iturin at 450 mg/L, while CSC-6 led in fengycin production at 710 mg/L. The isolation of such native, wild-type strains that naturally accumulate lipopeptides at gram-per-liter levels is a significant finding. These strains therefore serve as excellent chassis both for direct fermentation-based bioproduct generation and as genetic reservoirs for future pathway engineering and heterologous expression studies aimed at further yield enhancement or novel lipopeptide design.

### 3.4 Lipopeptides extracts possess potent antimicrobial activity

To validate the functional relevance of the high lipopeptide yields, we evaluated the antimicrobial activity of crude extracts from the most productive strains against the human pathogen *S. aureus* and the key plant pathogen *R. solani*. The results unequivocally demonstrated that the produced lipopeptides were biologically active and potent (Figures 1e,f). All lipopeptide extracts formed clear inhibition zones against *S. aureus*, with the high-surfactin producer A1C-8 exhibiting the strongest antibacterial activity at a 54.6% inhibition rate. More remarkably, the extracts displayed even greater efficacy against the filamentous fungus *R. solani*. The A1C-8 extract achieved a remarkable 273.3% inhibition rate, and the Bv1 extract 213.3%. This exceptional antifungal activity likely stems from the combined action of the different lipopeptide families present in the crude extracts. This broad-spectrum antimicrobial activity demonstrates the extensive application potential of lipopeptides in agriculture and medicine. While we prioritized agricultural applications in follow-up studies, the potent activity against *S. aureus* also establishes them as promising candidates for addressing antibiotic resistance and life-threatening infections.

### 3.5 Identification of the synthetic genes for antimicrobial peptides in antagonistic bacteria

**Figure 3:**
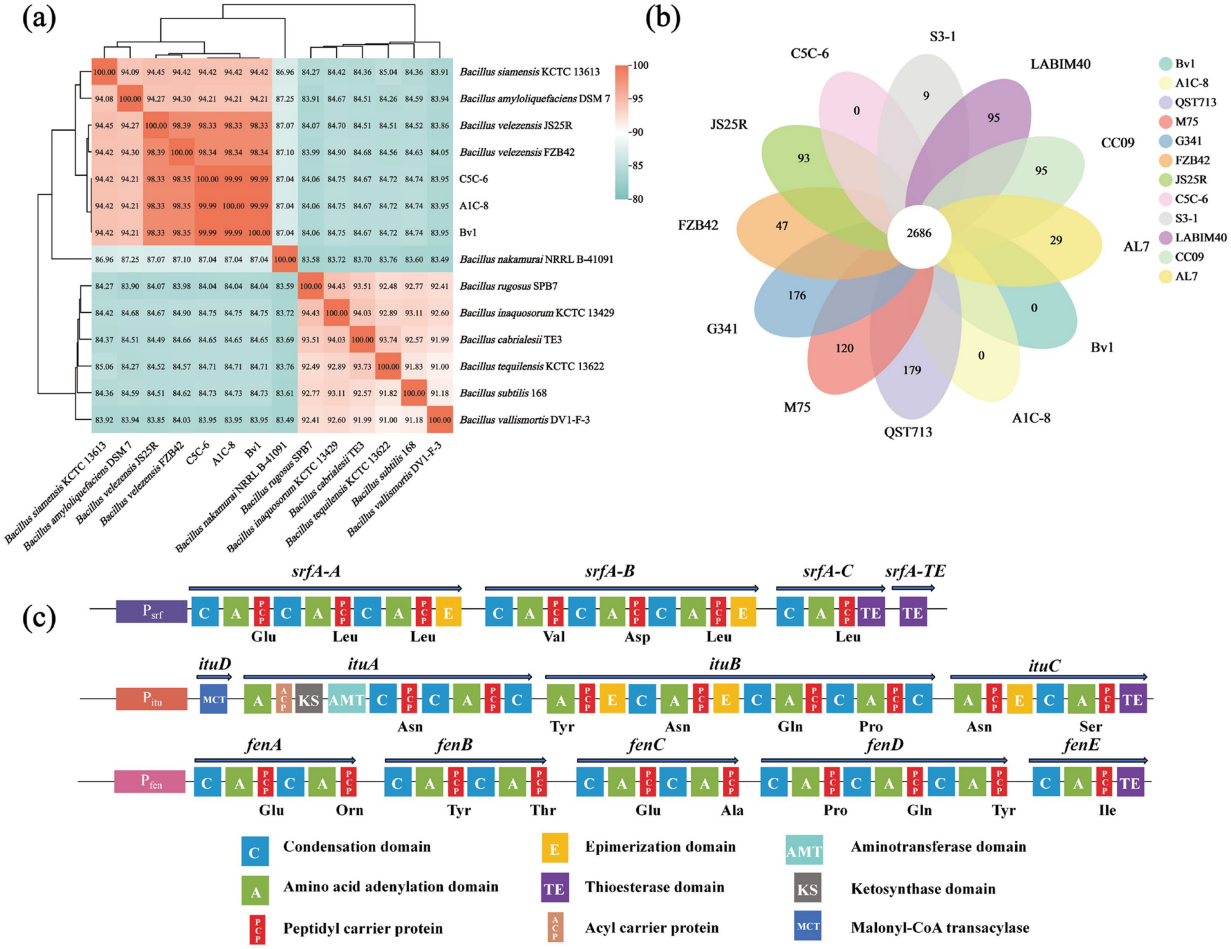
Genomic features and lipopeptide potential of the isolated *Bacillus* strains. **(a)** Heatmap of ANI values among different strains of *Bacillus* strains. **(b)** Number of specific genes in each *B. velezensis* strain. The inner circle represents the core genome shared by all strains. **(c)** Genomic organization and predicted structures of the lipopeptide biosynthesis gene clusters (surfactin, iturin, and fengycin) in the genomes of Bv1, A1C-8, and C5C-6.

To elucidate the genetic foundation of the observed high lipopeptide production and multifunctionality, the whole genomes of the top-producing strains, A1C-8, CSC-6, and Bv1. The general genomic features of these strains are summarized in Supplementary Table S3, and their circular genome maps are visually presented in Supplementary Figure S4. The ANI values of these strains against all *B. velezensis* strains were >98%, which is above the established threshold of 96% (Figure 3a) (Richter and Rosselló-Móra, 2009). As anticipated, bioinformatics mining using antiSMASH revealed the presence of complete and intact nonribosomal peptide synthetase (NRPS) gene clusters responsible for the biosynthesis of surfactin (*srf*), iturin (*itu*), and fengycin (*fen*) in all three genomes (Figure 3c). The identification of these clusters provides direct genetic validation of our phenotype-based screening strategy. An interesting observation was the lack of fengycin detection via MALDI-TOF MS in strain A1C-8 despite the presence of the corresponding gene cluster. This underscores the strong post-genomic regulation of lipopeptide synthesis, where environmental cues, fermentation conditions, and complex regulatory networks ultimately determine which clusters are actively expressed and at what level, highlighting the importance of optimizing culture parameters for metabolite production (Sun et al., 2019).

To contextualize our isolates within the *B. velezensis* species, we conducted a pan-genome analysis with nine other publicly available genomes downloaded from NCBI Genbank database. The analysis revealed a core genome of 2,686 genes, indicative of a highly conserved genetic backbone essential for basic physiology and the hallmark *B. velezensis* lifestyle (Figure 3b). Notably, the pan-genome size showed an unbounded increase with the addition of new genomes (open pan-genome), while the core genome stabilized (Supplementary Figure S5). This pattern, characteristic of species with diverse lifestyles and niche adaptations, suggests *B. velezensis* has a high capacity for horizontal gene transfer and genetic innovation.

### 3.6 Lipopeptide extracts directly enhance crop growth and yield in field trials

#### 3.6.1 Lipopeptide extracts enhance maize growth and yield

**Figure 4:**
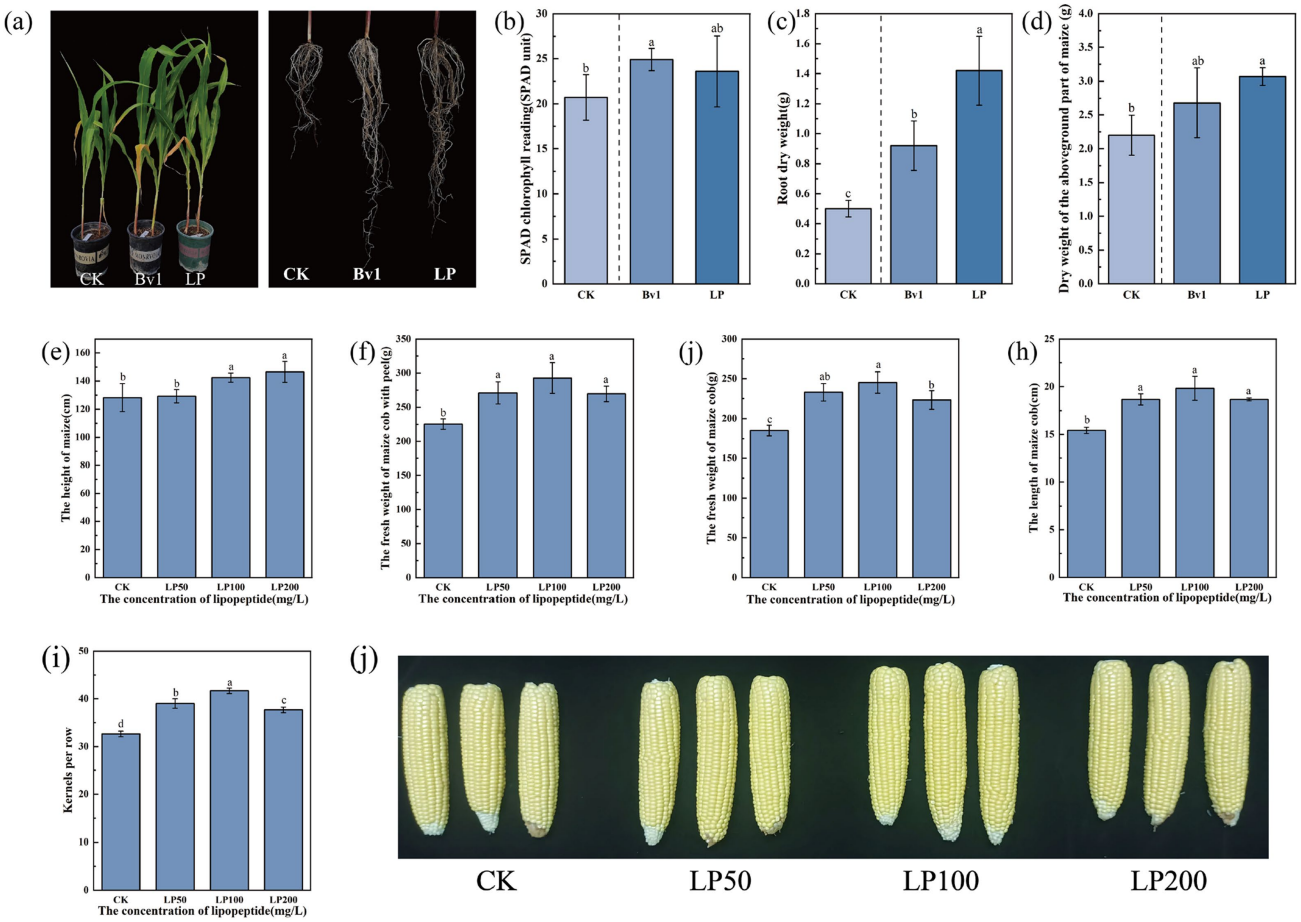
Effects of bacterial inoculation and root irrigation with lipopeptide on maize growth and yield. **(a)** Representative potted plants and corresponding root morphology of seedlings at 27 days after sowing. **(b–d)** Seedling traits at 27 days: **(b)** leaf chlorophyll content (SPAD value), **(c)** root dry weight, and **(d)** shoot dry weight. **(e–i)** Field performance and yield components at maturity: **(e)** plant height at the heading stage, **(f)** fresh weight of cob with husk, **(g)** fresh weight of debusked cob, **(h)** cob length, and **(i)** number of kernels per row. **(j)** Representative photographs of maize cobs from the different treatment groups at harvest.

Maize, a cornerstone of global cereal production, was selected for initial pot trials using strain Bv1, chosen for its balanced high production of surfactin, iturin, and fengycin coupled with pronounced PGPR traits. After 20 days of treatment, significant growth promotion was observed in plants treated with either the lipopeptide extract or the lyophilized Bv1 powder compared to the control (Figure 4a). The most dramatic effect was recorded in root system development. Root dry weight increased by 185.91 and 84.56% in the lipopeptide extract and bacterial powder treatments, respectively (Figure 4c). Both the extract and bacterial treatments significantly increased plant growth. The extract increased aboveground dry weight by 39.76% and leaf chlorophyll content (SPAD) by 14.09%, while the bacteria increased them by 22.15 and 20.05%, respectively (Figures 4b,d). Additional growth-promoting parameters are presented in Supplementary Figure S7. Notably, these positive effects were rapid; significant improvements in plant height, stem thickness, and chlorophyll content were evident as early as 10 days post-treatment and persisted throughout the experiment (Supplementary Figure S6). This consistent promotion suggests a sustained physiological effect rather than a transient response.

To transition from controlled pot conditions to field relevance, a concentration-gradient field trial was conducted. At the jointing stage, soil application of the lipopeptide at 100 and 200 mg/L significantly increased plant height by 11.1 and 14.2%, respectively, compared to the untreated control (128.23 ± 9.98 cm) (*p* < 0.05; Figure 4e). By the milk-ripening stage, all lipopeptide treatments improved ear productivity, with the 100 mg/L concentration consistently showing the greatest effect (Figure 4j). This treatment increased fresh ear weight (with husk) by 29.91%, dehusked fresh ear weight by 32.46%, ear length by 28.51%, and the number of kernels per row by 27.55% relative to the control (Figures 4f–i). The 50 mg/L and 200 mg/L treatments also significantly enhanced these yield components, though to a lesser extent, indicating a non-linear, optimal concentration response curve. The enhancement of kernels per row is particularly significant, as it is a major determinant of final grain yield. These results collectively demonstrate that lipopeptide application, particularly at an optimized concentration of 100 mg/L, serves as an effective strategy to enhance the productivity of field-grown maize by improving crucial yield-architecture traits.

#### 3.6.2 A field assessment of lipopeptide-mediated growth promotion in peanut

**Figure 5:**
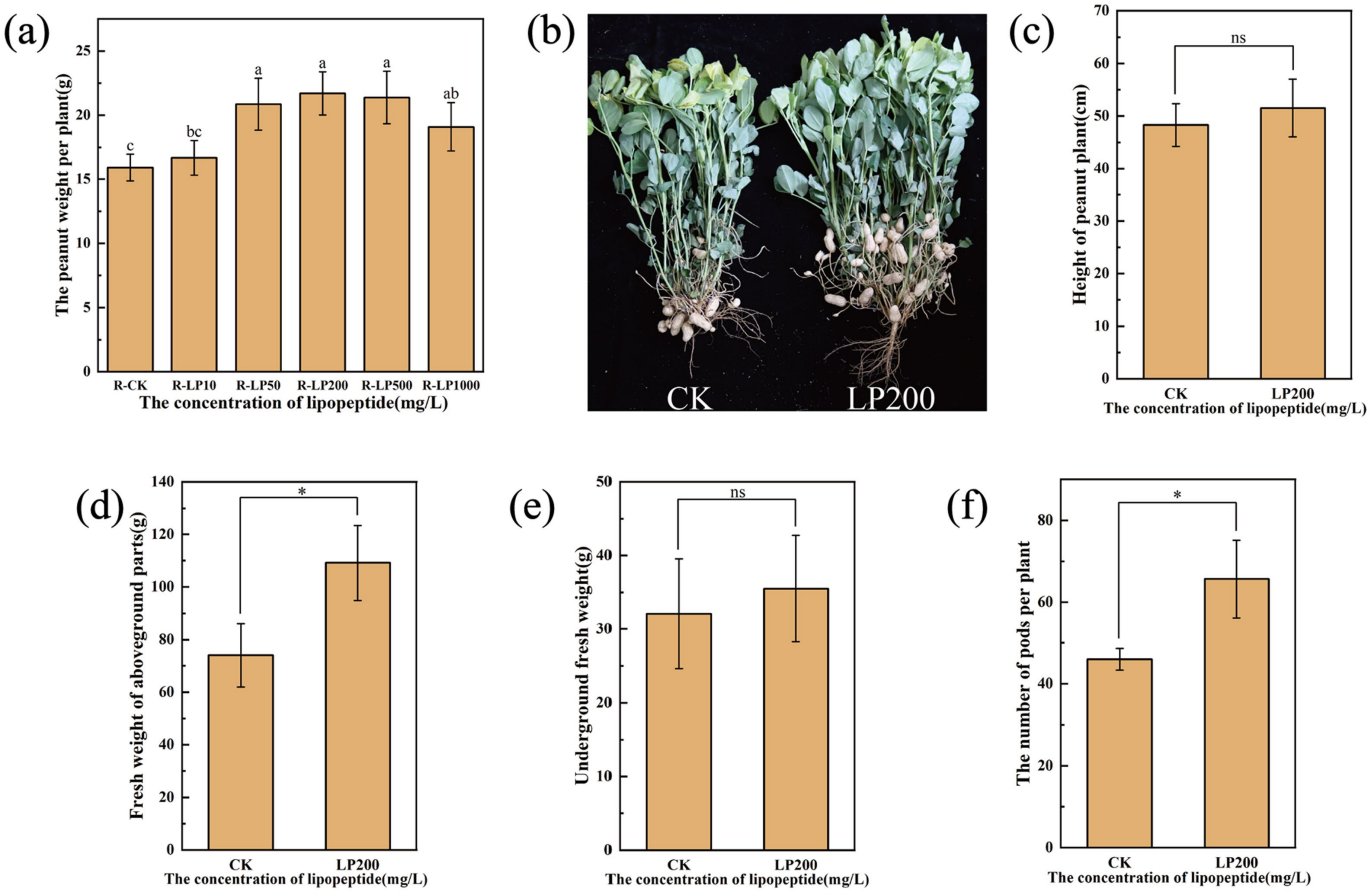
Effects of lipopeptide treatments on peanut growth and yield. **(a)** The peanut weight per plant under different lipopeptide concentrations. **(b–f)** A comparison of control and 200 mg/L lipopeptide-treated plants, showing: **(b)** A representative photograph, **(c)** plant height, **(d)** aboveground parts fresh weight, **(e)** underground parts fresh weight, and **(f)** pod number per plant.

Peanut presents a unique case study due to its reproductive process involving underground pod development, known as geocarpy (Liew et al., 2021). We first aimed to identify the optimal lipopeptide concentration for yield enhancement. A broad range of concentrations (10–1,000 mg/L) was tested. Remarkably, all concentrations significantly increased yield per plant compared to the control, confirming the robust growth-promoting activity (Figure 5a). The effect was concentration-dependent, with 200 mg/L providing the most substantial boost (36.49% increase). Significant yield promotions of 31.21, 34.45, and 20.08% were also achieved with 50, 500, and 1,000 mg/L treatments, respectively, while the lowest concentration (10 mg/L) resulted in a modest 4.81% increase. The apparent decrease in efficacy at very high concentrations (500–1,000 mg/L) warrants further investigation but may relate to potential phytostatic effects or altered soil microbial dynamics at excessive doses.

Based on this screening, the 200 mg/L concentration was selected for detailed field evaluation. Soil drenching at the seedling stage significantly improved multiple agronomic parameters (Figure 5b). Lipopeptide treatment increased plant height by 6.69%, aboveground fresh weight by 47.5%, and underground fresh weight (including roots and developing pods) by 10.59% (Figures 5c–e). Most importantly, the pod number per plant, a direct yield component, rose dramatically by 42.75% (Figure 5f). Subsequent analysis of harvested pods confirmed the yield enhancement: pod weight per plant increased by 18.6% (Supplementary Table S4). Furthermore, quality parameters also improved, with the plump-kernel rate showing a significant 7.3% increase, and the double-pod rate, 100-pod weight, and 100-kernel weight rising by 2.9, 2.7, and 6.4%, respectively. This dual effect on both yield quantity and quality underscores the potential economic value of lipopeptide application in legume production.

#### 3.6.3 Lipopeptide extracts enhance sunflower growth and yield

**Figure 6:**
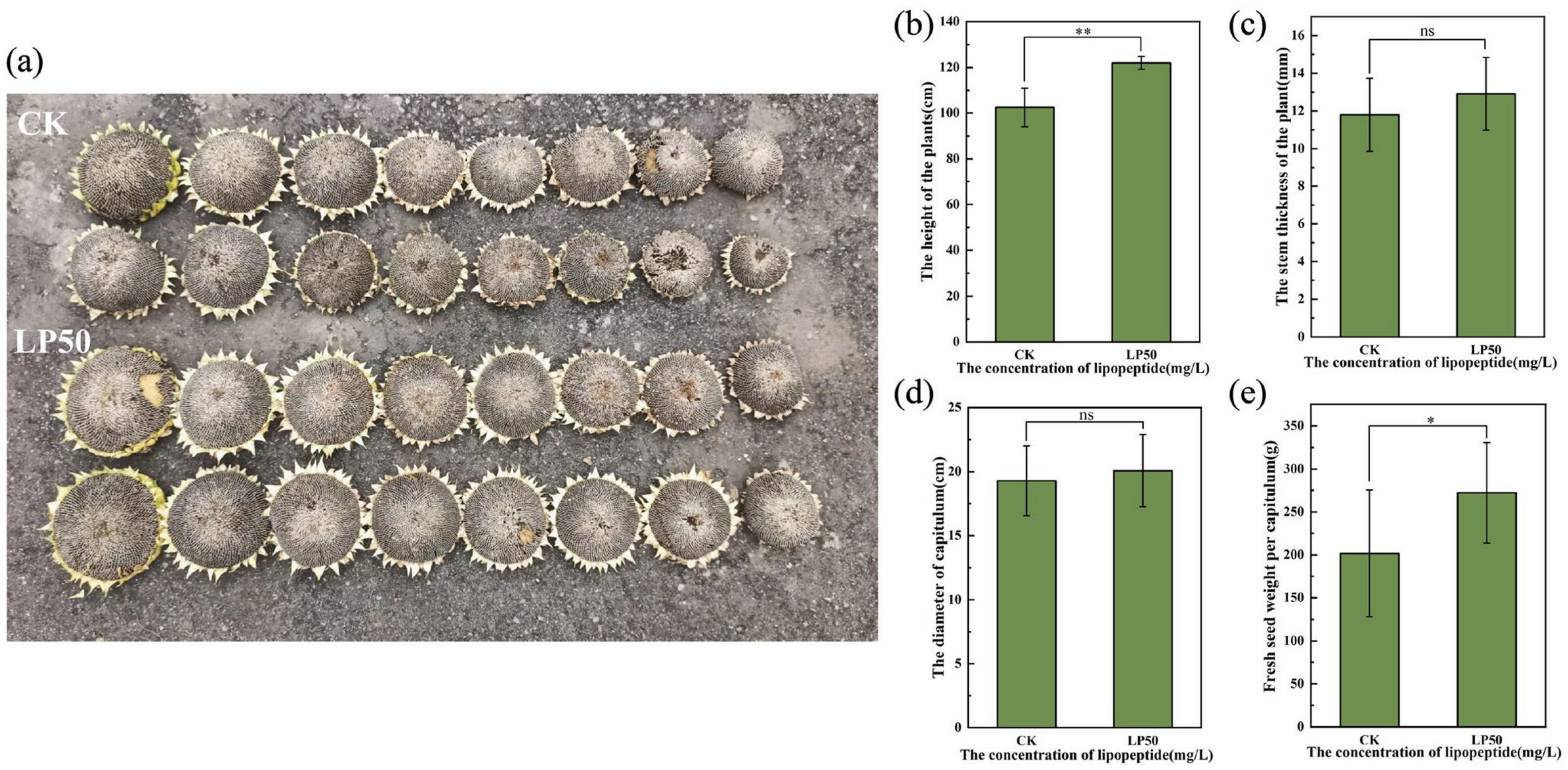
Effects of lipopeptide treatment on sunflower traits at harvest. **(a)** Representative image of sunflower capitulum. **(b–e)** A comparison between control and 50 mg/L lipopeptide-treated plants, with panels showing: **(b)** Plant height, **(c)** Stem diameter, **(d)** Head diameter, and **(e)** Fresh seed weight per head.

To assess the broad-spectrum applicability of our findings, we evaluated lipopeptide effects on sunflower, a major global oilseed crop. Under field conditions, application of 50 mg/L lipopeptide extract significantly promoted vegetative growth. At 24 days after treatment, plant height showed a marked increase of 19.02%, and stem diameter was enhanced by 9.46% compared to the control (Figures 6b,c). This stronger stem architecture could contribute to improved lodging resistance. At physiological maturity (92 days), treated sunflowers exhibited improved yield-related traits. The capitulum (flower head) diameter increased by 4.15% (Figures 6a,d). Crucially, the fresh seed weight per capitulum, the primary economic yield parameter, was 35% higher in the lipopeptide-treated group (Figure 6e). For oilseed crops, seed weight is directly correlated with oil yield, making this finding particularly relevant for bioresource and bioenergy applications.

## 4 Discussion

The development of effective and consistent microbial inoculants for sustainable agriculture faces two principal challenges. One challenge is the scarcity of multifunctional, competitive rhizosphere strains that deliver key plant-beneficial services, a problem magnified by the low proportion (1–5%) of culturable soil microbes (Hakim et al., 2021). Another is the industrial scalability of their bioactive metabolites, particularly lipopeptides, which are often produced in low yields by wild-type strains, a key limiting factor that has been consistently identified as a major barrier to their widespread commercial application (Farooq et al., 2025). To address these challenges, we designed a tiered screening strategy aimed at discovering native *Bacillus* strains with both antagonistic and plant-growth-promoting traits, and identifying elite isolates with high lipopeptide production capacity. The sequential integration of hemolysis, antagonism, and oil-spreading assays funnels the screening from a broad search to the refined selection of high-yielding strains, significantly enhancing discovery efficiency and success (Mnif and Ghribi, 2015; Cawoy et al., 2015). This approach incorporates the well-established dose-dependent hemolytic activity of iturin-family compounds, such as iturin A, on erythrocyte membranes (Aranda et al., 2005). The efficacy of this screening strategy was rigorously validated by MALDI-TOF MS. All candidate strains selected were confirmed to produce diverse lipopeptides, thereby effectively excluding false-positive results (Table 1). HPLC quantification further validated the screening efficacy, with selected strains producing surfactin (1,140 mg/L), iturin (450 mg/L), and fengycin (710 mg/L) under unoptimized shake-flask conditions (Figure 1d).

For instance, the lipopeptide-high-producing strain *B. amyloliquefaciens* HM618 produced 0.724 g/L of surfactin under unoptimized medium in pure culture (Liu et al., 2023). The wild-type strain *B. amyloliquefaciens* HZ-12 produced approximately 0.43 g/L of iturin A in shake flasks, and its engineered derivative HZ-T14 reached 8.53 g/L, which is the highest iturin A yield reported so far (She et al., 2024). In *Bacillus subtilis* CGF-P-02, engineered to overexpress the proline-transporter gene *opuE* and supplemented with 8.0 g L^−1^ exogenous proline, fengycin production reached 871.86 mg/L (Gao et al., 2023). Therefore, at the initial screening stage, the wild-type strain we isolated demonstrated superior lipopeptide production compared to the high-yielding strains reported above, with a titer several-fold greater than that of many other wild-type *Bacillus* strains. These strains thus function as versatile platforms, suitable both for direct lipopeptide fermentation and as genetic repositories for future engineering aimed at yield improvement or novel molecule design.

Successful rhizosphere inhabitants, such as plant growth promoting rhizobacteria (PGPR), employ diverse mechanisms rather than relying on a single function to establish their role (Prashar et al., 2014). These strains employ a versatile array of traits that act synergistically to sustain plant performance under fluctuating edaphic conditions (Kerbab et al., 2021). We therefore incorporated assays for siderophore production, IAA yield, protease activity, and phosphate solubilization ability. Notably, all candidate strains isolated in this study possessed a complete suite of these traits (Figure 1c). The concurrent presence of these PGPR-associated traits in our high-yielding lipopeptide producers suggests a strong synergistic mode of action, positioning them as multifunctional candidates for plant biostimulation and biocontrol. These strains can directly antagonize pathogens via lipopeptides, suppress them through siderophore-mediated competition and protease activity, and simultaneously enhance plant growth via IAA and improved phosphate nutrition. This multifunctionality is critical for rhizosphere competence and resilience (Huang et al., 2023), making these strains ideal candidates not only as standalone inoculants but also as core components in designed synthetic microbial consortia, where complementary traits can be combined for more robust and predictable outcomes in diverse agricultural settings.

Key to the lipopeptide production in our elite strains was the presence of nonribosomal peptide synthetase (NRPS) gene clusters within their genomes (Figure 2c). These gene clusters encode the corresponding NRPSs, which catalyze lipopeptide synthesis via a modular assembly-line mechanism. Each module typically comprises adenylation (A), thiolation (PCP), and condensation (C) domains, which collectively function to select, activate, and incorporate a specific amino acid into the elongating peptide chain (Mootz et al., 2002). The number, type, and sequential order of these modules dictate the amino acid identity, sequence, and the length of the resultant peptide chain (Ishikawa et al., 2024). The heterologous expression of NRPS gene clusters remains challenging due to their large size and structural complexity, as exemplified by the iturin A operon, which spans more than 38 kb (Tsuge et al., 2001). Although the recently developed CTCNT-P technique provides an efficient and universal method for genetic manipulation in *Bacillus*, its efficiency remains constrained by specific factors such as the natural competence of the recipient strain and plasmid type (Wang et al., 2025). The electroporation of undomesticated *Bacillus* strains presents a significant challenge, often yielding zero transformants with standard protocols, thus requiring systematic optimization of conditions to achieve any measurable efficiency (Zhao et al., 2024). Therefore, systematic screening for high-yielding *Bacillus* strains remains essential to provide robust industrial production strains and to generate the genomic resources required for subsequent genetic engineering. Furthermore, the presence of large, complex NRPS clusters in these naturally high-yielding strains provides a valuable genetic resource for fundamental studies on lipopeptide biosynthesis regulation and for advanced metabolic engineering efforts.

Field trials with the selected strain demonstrated that both its bacterial cells and the lipopeptide extract significantly promoted the growth and yield of maize, peanut, and sunflower, thereby completing the cycle of translational research from the laboratory to the field. In contrast to prior studies emphasizing the biocontrol capacity of lipopeptides via induced systemic resistance (ISR) (Ongena et al., 2007; Rodríguez et al., 2018; Ding et al., 2025), our work highlights their underexplored role as direct plant biostimulants, consistently increasing the vegetative biomass and marketable yield of these three key crop families under natural field conditions. The profound root stimulation observed is of paramount agronomic importance, as an expanded root architecture enhances water and nutrient foraging, potentially increasing drought tolerance and fertilizer use efficiency (Shoaib et al., 2022; Yu et al., 2024). The observed growth promotion may be mechanistically linked to surfactin, which enhances bacterial motility by upregulating flagellin gene expression, thereby enabling more robust colonization of surfaces such as plant roots by beneficial soil or inoculant bacteria (Ghelardi et al., 2012). Furthermore, the biosurfactant nature of these compounds could improve the wettability and permeability of root surfaces, facilitating nutrient uptake (Silva et al., 2024). Regarding the specific yield components, the dramatic increase in peanut pod number suggests that lipopeptides may influence critical reproductive stages such as flowering, peg formation, or the peg-to-pod transition, with the concurrent improvement in kernel plumpness indicating enhanced photoassimilate partitioning and pod filling. In summary, our findings reveal an additional, direct plant-biostimulatory function of *Bacillus* lipopeptides beyond their established biocontrol capacity, a role that translates into tangible yield gains across diverse crops.

## 5 Conclusion and future perspectives

A tiered functional screening strategy successfully isolated high-yielding lipopeptide-producing *Bacillus velezensis* strains from rhizosphere soils. These strains possessed robust biosynthetic gene clusters and produced gram-per-liter levels of lipopeptides (surfactin, iturin, and fengycin), demonstrating potent broad-spectrum antimicrobial activity. Crucially, field validation revealed a previously underexplored, direct plant biostimulant function: crude lipopeptide extracts significantly enhanced growth and yield in maize, peanut, and sunflower. This work establishes a complete pipeline from strain discovery to field application, positioning these lipopeptides as sustainable, dual-function agents for biocontrol and growth promotion, offering a viable alternative to synthetic agrochemicals.

However, whether the observed growth promotion is attributable to lipopeptide production and the exact mechanism by which lipopeptides themselves directly stimulate plant growth remain elusive and require further investigation. Future studies could integrate dual RNA-seq transcriptomics with targeted phytohormone profiling and reverse-genetics validation to delineate the lipopeptide-activated signaling cascades underlying growth promotion.

## Data Availability

The datasets presented in this study can be found in online repositories. The names of the repository/repositories and accession number(s) can be found in the article/Supplementary material.
