# Supplementary material for: From soil to crop: a tiered screening strategy for lipopeptide-producing Bacillus with antifungal, antibacterial and plant growth-promoting activities
Source: Front Microbiol. 2026 Apr 28;17:1798265. doi: 10.3389/fmicb.2026.1798265 (PMC13161030; doi:10.3389/fmicb.2026.1798265)
Supplement: Supplementary file 1 [file Table_1.DOCX]

Supplementary Material

Supplementary Table 1. Details of soil sample collection location.

| Sampling location (Province and city) | Soil sample area | Latitude and longitude | Isolated strains |
| --- | --- | --- | --- |
| Chengdu, Sichuan Province | Radish field | 103°46’E 30°41’N | A1C-6, A1C-8 |
| Xi'an City, Shaanxi Province | Greenbelt soil | 108°93’E 34°23’N | A3B-6 |
| Yueyang City, Hunan Province | Vegetable field | 112°90’E 28°68’N | A5A-3 |
| Lijiang City, Yunnan Province | Apple tree rhizosphere soil | 100°25’E 26°86’N | A5C-14 |
| Changping District, Beijing | Soybean field | 116°25’E 40°7’N | A6A-1 |
| Xiantao City, Hubei Province | Radish field | 112°20’E 30°18’N | A6B-5 |
| Xi'an City, Shaanxi Province | Greenbelt soil | 108°58’E 34°19’N | A6C-9 |
| Changde City, Hunan Province | Garlic scallion field | 111°60’E 28°87’N | A6D-11 |
| Liaoyang City, Liaoning Province | Cabbage field | 123°22’E 41°37’N | B1B-5 |
| Duyun City, Guizhou Province | Wheat field | 107°65’E 26°07’N | B2D-3 |
| Haikou City, Hainan Province | Cabbage field | 110°01’E 20°00’N | C1C-2 |
| Datong City, Shanxi Province | Rhizosphere soil of Poplar | 113°20’E 40°3’N | C5A-1 |
| Hangzhou City, Zhejiang Province | Rhizosphere soil of tea plant | 120°10’E 30°22’N | C5C-6 |
| Pinggu District, Beijing | Tomato field | 117°15’E 40°18’N | Bv1 |

**
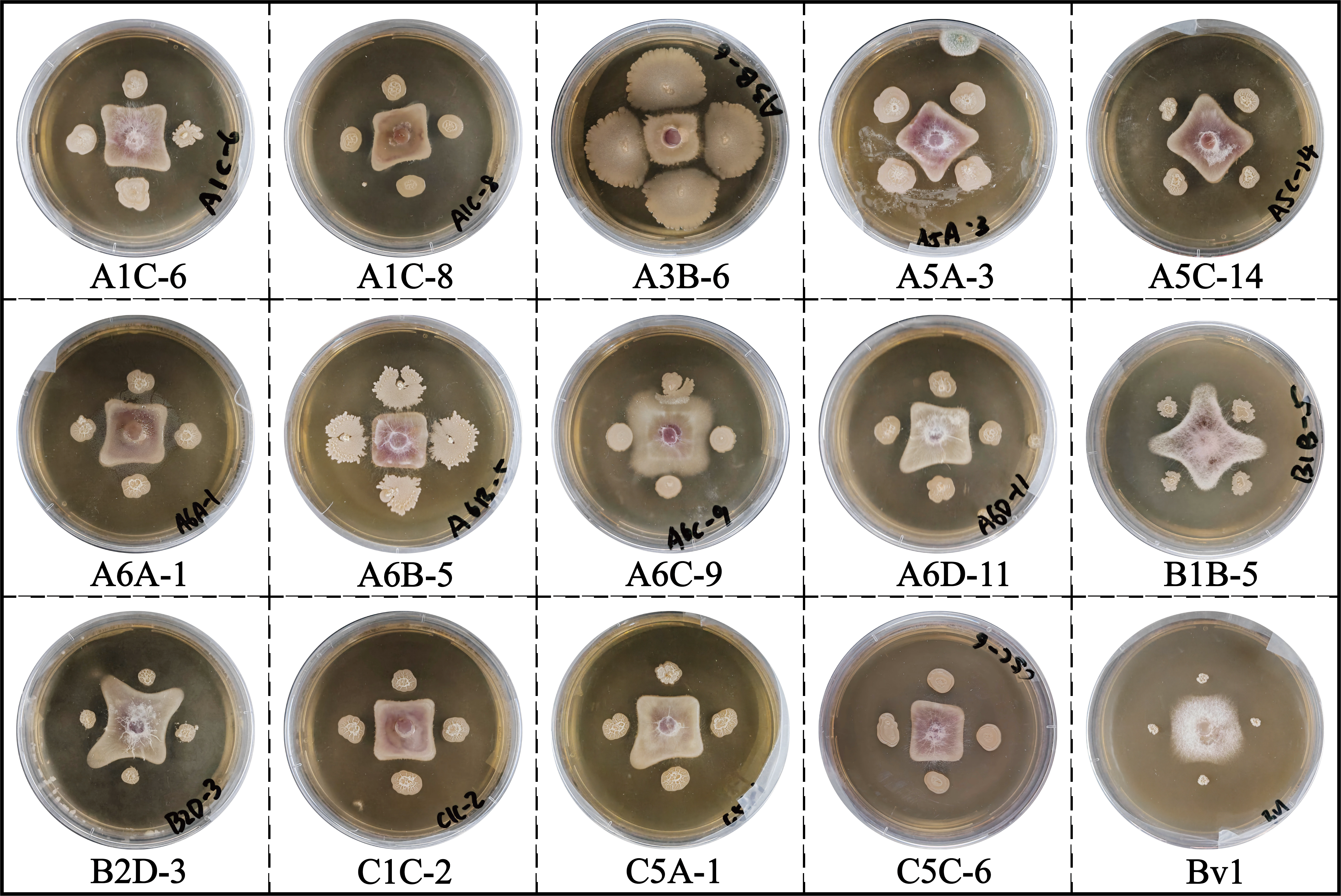
**

**Supplementary Figure 1.** Antagonistic activity of different *Bacillus* strains against *R. solani* in plate confrontation.


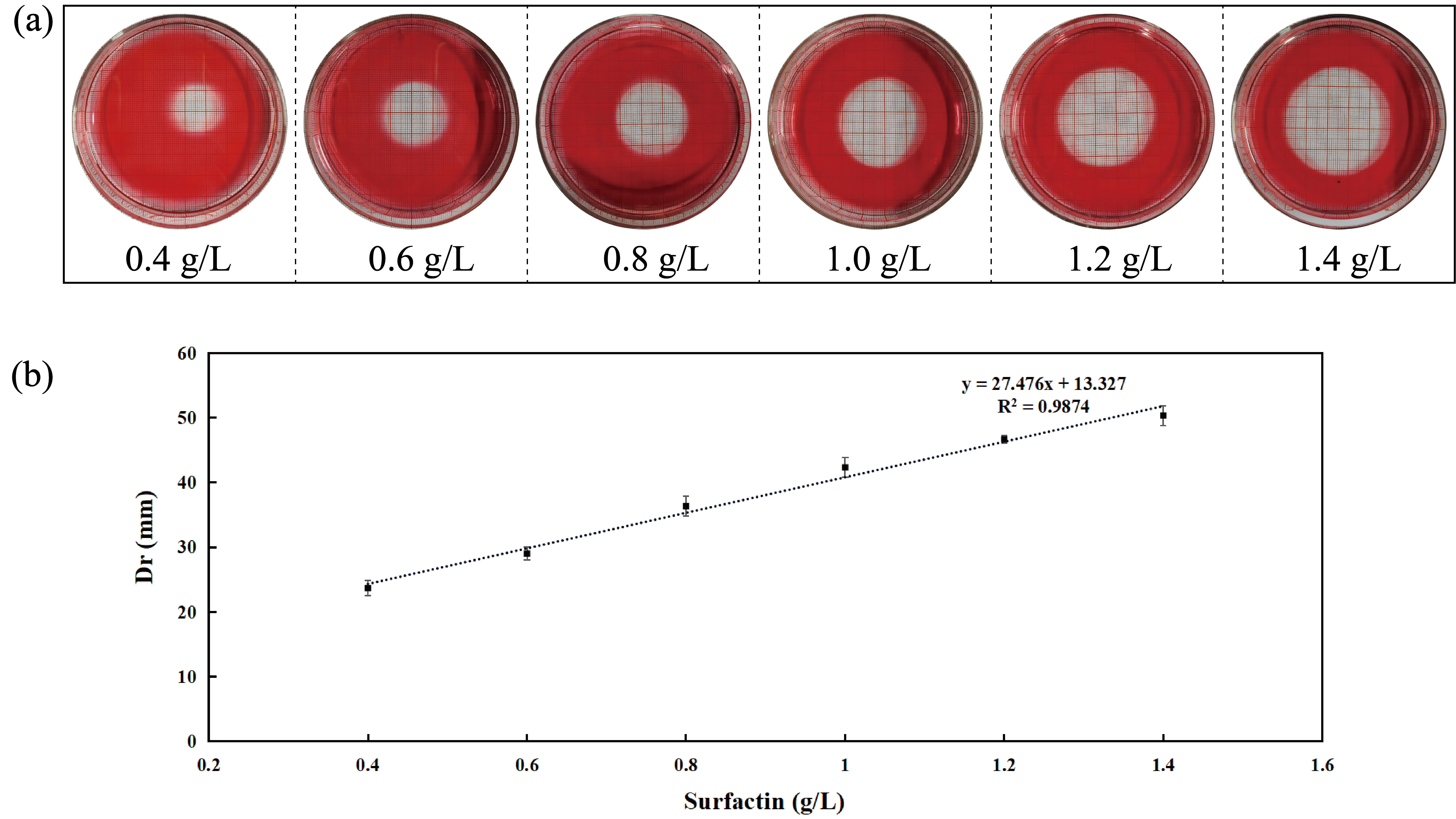


**Supplementary Figure 2.** Oil-spreading assay for surfactin quantification. (a) Clear zones produced by serial concentrations of surfactin standard. (b) Standard curve showing the linear correlation between the diameter of the clear zone and surfactin concentration.

Supplementary Table 2. Plant growth-promoting rhizobacteria (PGPR) traits of the isolated strains.

| Isolate | IAA (µg/mL) | Siderophore production | Phosphate solubilization | Protease |
| --- | --- | --- | --- | --- |
| A1C-6 | 9.29±0.65 | ++ | ++ | +++ |
| A1C-8 | 8.95±0.75 | ++ | ++ | +++ |
| A5A-3 | 8.81±0.30 | ++ | + | +++ |
| A5C-14 | 9.07±0.07 | ++ | ++ | +++ |
| A6A-1 | 7.97±0.30 | ++ | ++ | +++ |
| A6B-5 | 8.63±0.28 | ++ | ++ | + |
| A6D-11 | 9.62±0.30 | ++ | ++ | +++ |
| B2D-3 | 8.45±0.24 | ++ | ++ | +++ |
| Bv1 | 9.61±0.69 | ++ | +++ | +++ |
| C1C-2 | 8.83±0.41 | ++ | ++ | +++ |
| C5A-1 | 9.60±0.07 | +++ | +++ | +++ |
| C5C-6 | 9.94±0.07 | ++ | +++ | +++ |

*Note: The intensity of each trait was scored as: +, weak activity ; ++, moderate activity; +++, strong activity .*

*
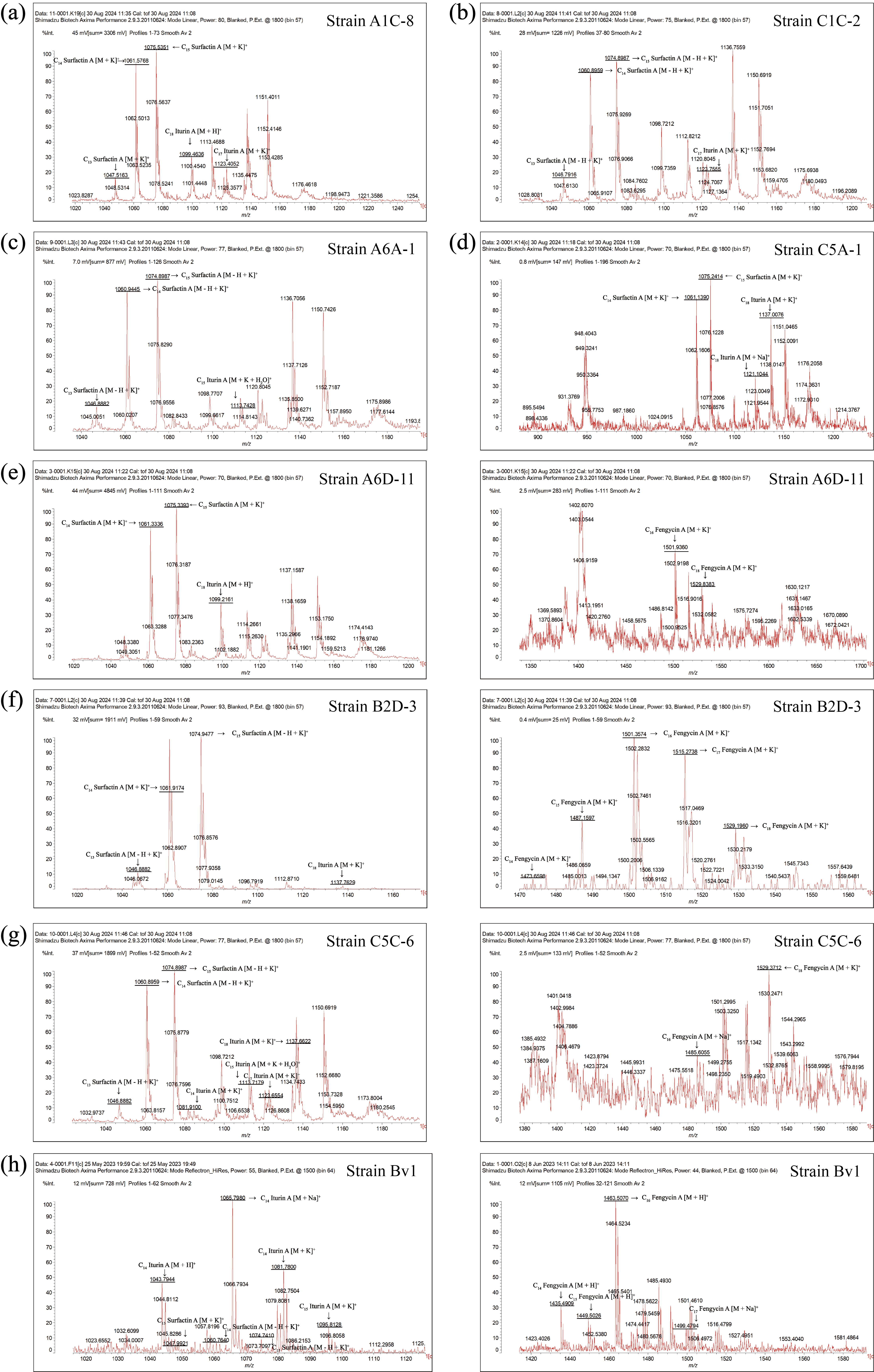
*

**Supplementary Figure 3.** MALDI-TOF MS analysis of lipopeptides produced by various bacterial isolates.

Supplementary Table 3. Genomic features of *B. velezensis* strains A1C-8, Bv1, and C5C-6.

| Issue | A1C-8 | Bv1 | C5C-6 |
| --- | --- | --- | --- |
| Genome size (bp) | 3929585 | 3929792 | 3929585 |
| GC content (%) | 46.5 | 46.5 | 46.5 |
| Protein-coding genes | 3746 | 3747 | 3746 |
| Gene total length (bp) | 3474915 | 3475596 | 3474948 |
| Gene average length (bp) | 927.63 | 927.57 | 927.64 |
| Gene length/genome | 88.43 | 88.44 | 88.43 |
| GC content in gene region (%) | 40.05 | 47.34 | 40.05 |
| tRNA number | 86 | 86 | 86 |
| rRNA number | 27 | 27 | 27 |
| Accession number | CP187372 | CP187375 | CP187373 |


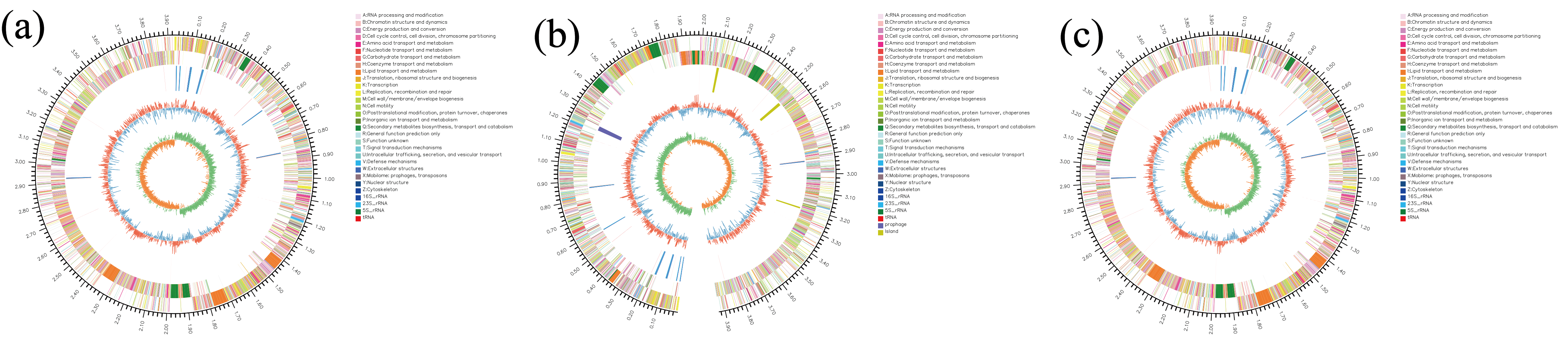


**Supplementary Figure 4.** Circular genome maps of *B. velezensis* strains. (a) A1C-8, (b) Bv1, and (c) C5C-6.


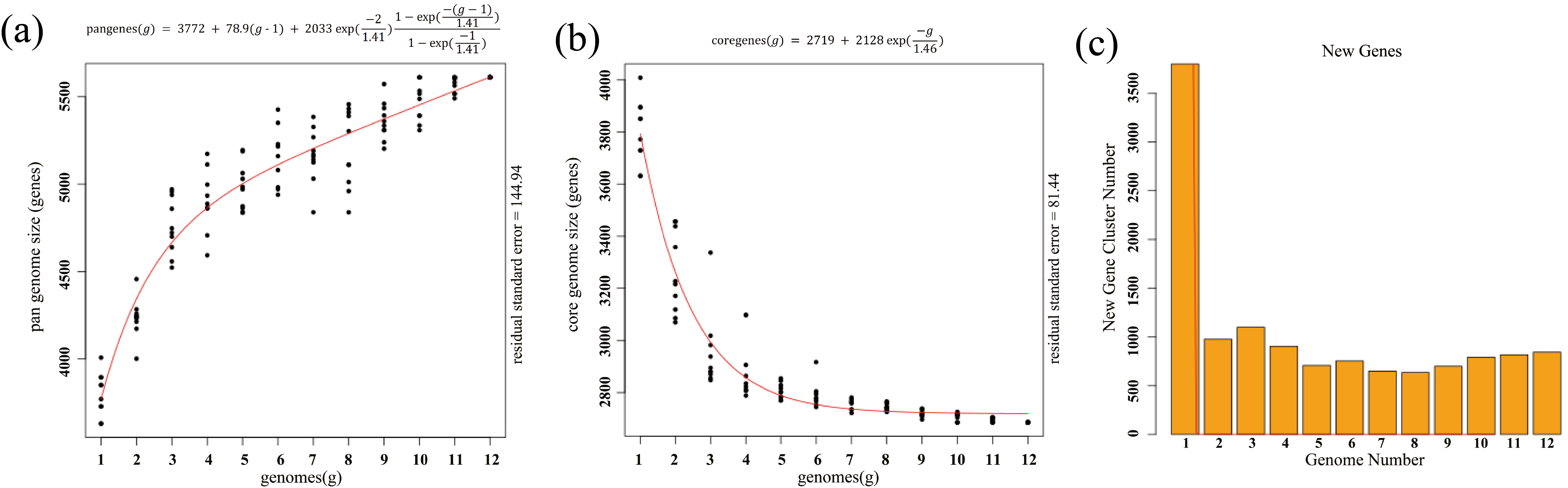


**Supplementary Figure 5.** Pan-genome analysis curves of *B. velezensis* strains. (a) Pan-genome curve. (b) Core-genome curve. (c) Curve of novel gene cluster discovery rate relative to the number of genomes analyzed.


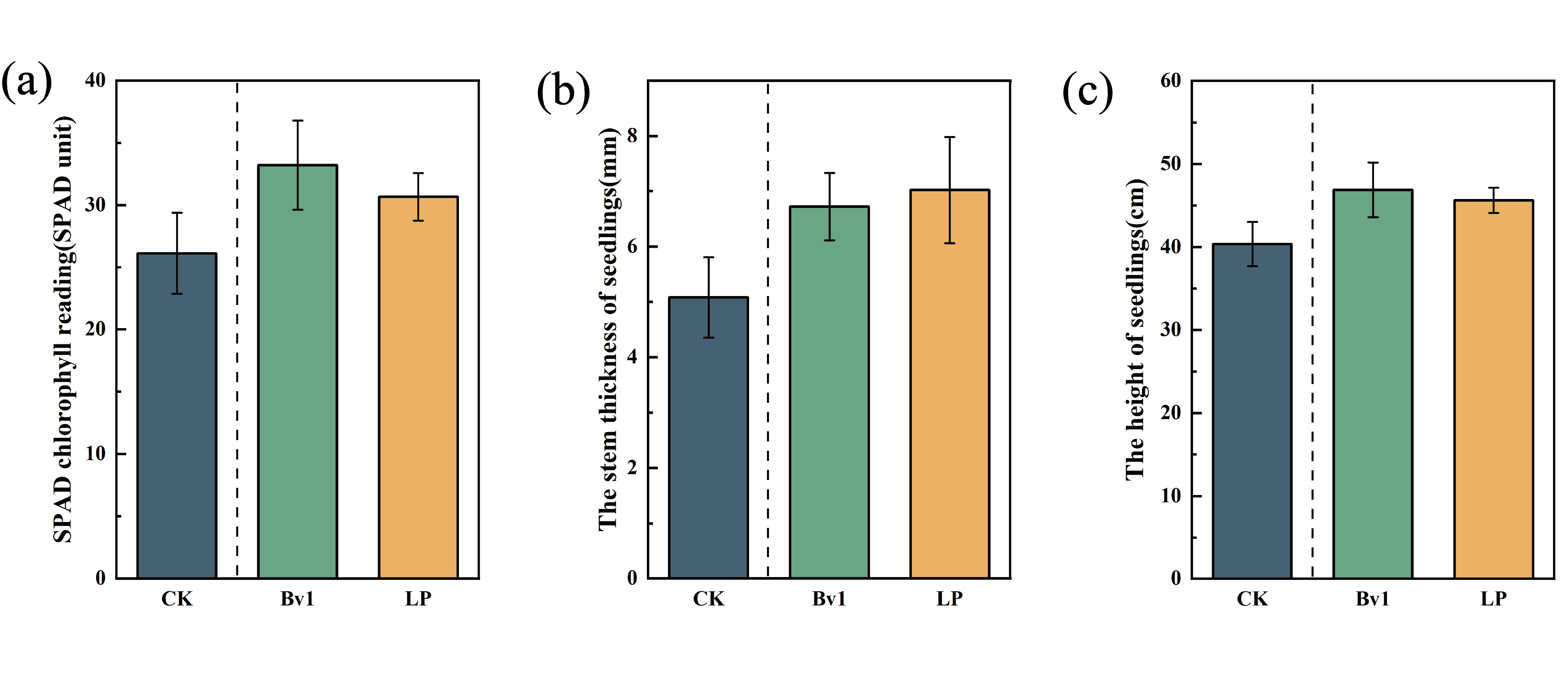


**Supplementary Figure 6.** Maize growth parameters at 10 days after treatment. (a) Chlorophyll content. (b) Stem thickness. (c) Plant height.


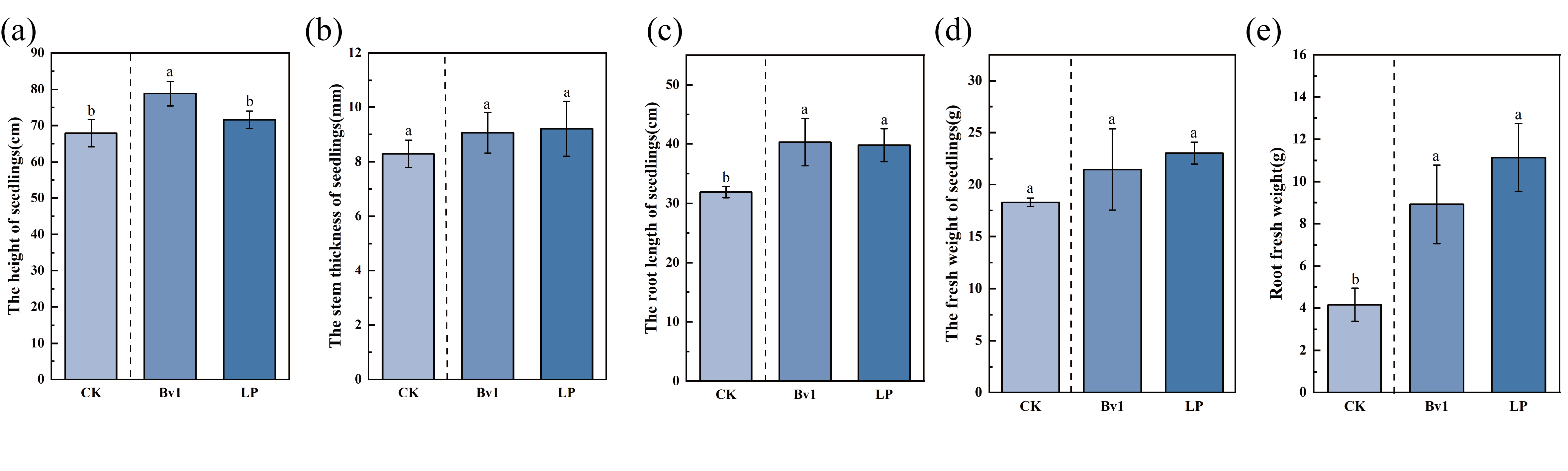


**Supplementary Figure 7.** Assessment of maize seedling growth promotion at 27 days after sowing. (a) The height of seedlings. (b) The stem thickness of seedlings. (c) The root length of seedlings. (d) The fresh weight of seedlings. (e) Root fresh weight.

Supplementary Table 4. Effects of lipopeptide treatments on peanut yield components and quality parameters. Values are presented as mean ± standard deviation (SD; n=3). *Significantly different from control (p < 0.05).

| Treatment (mg/L) | Pod yield per plant (g) | double-pod rate  (%) | Plump kernel rate (%) | 100-pod weight (g) | 100-kernel weight (g) |
| --- | --- | --- | --- | --- | --- |
| 0 (CK) | 11.27±1.12 | 71.35±3.17 | 87.15±2.94 | 98.81±2.72 | 43.63±1.03 |
| 200 (LP) | 13.37±1.74 | 73.42±2.26 | 93.51±2.40^*^ | 101.42±3.75 | 46.42±1.95 |
